# Strategies to increase the activity of microglia as efficient protectors of the brain against infections

**DOI:** 10.3389/fncel.2014.00138

**Published:** 2014-05-22

**Authors:** Roland Nau, Sandra Ribes, Marija Djukic, Helmut Eiffert

**Affiliations:** ^1^Department of Neuropathology, University Medical Centre GöttingenGöttingen, Germany; ^2^Department of Geriatrics, Evangelisches Krankenhaus Göttingen-WeendeGöttingen, Germany; ^3^Department of Clinical Microbiology, University Medical Centre GöttingenGöttingen, Germany

**Keywords:** microglia, blood–brain barrier, blood–CSF barrier, innate immune system, bacteria, phagocytosis, palmitoylethanolamide

## Abstract

In healthy individuals, infections of the central nervous system (CNS) are comparatively rare. Based on the ability of microglial cells to phagocytose and kill pathogens and on clinical findings in immunocompromised patients with CNS infections, we hypothesize that an intact microglial function is crucial to protect the brain from infections. Phagocytosis of pathogens by microglial cells can be stimulated by agonists of receptors of the innate immune system. Enhancing this pathway to increase the resistance of the brain to infections entails the risk of inducing collateral damage to the nervous tissue. The diversity of microglial cells opens avenue to selectively stimulate sub-populations responsible for the defence against pathogens without stimulating sub-populations which are responsible for collateral damage to the nervous tissue. Palmitoylethanolamide (PEA), an endogenous lipid, increased phagocytosis of bacteria by microglial cells *in vitro* without a measurable proinflammatory effect. It was tested clinically apparently without severe side effects. Glatiramer acetate increased phagocytosis of latex beads by microglia and monocytes, and dimethyl fumarate enhanced elimination of human immunodeficiency virus from infected macrophages without inducing a release of proinflammatory compounds. Therefore, the discovery of compounds which stimulate the elimination of pathogens without collateral damage of neuronal structures appears an achievable goal. PEA and, with limitations, glatiramer acetate and dimethyl fumarate appear promising candidates.

## INTRODUCTION

In healthy young and middle-aged individuals beyond the neonatal period, infections of the central nervous system (CNS) are rare events. Although the blood–brain and blood–cerebrospinal fluid (CSF) barriers, i.e., the endothelium of the vessels situated in the brain parenchyma and the epithelium of the choroid plexuses, strongly impede the entry of pathogens into the CNS, it probably is not uncommon that pathogens reach the brain tissue or the CSF. In the CSF space, meningeal and perivascular and few circulating macrophages can eliminate pathogens (Engelhardt and Coisne, 2011). Moreover, pathogens can be transported by the CSF bulk flow (in humans approx. 20 ml/h) either through the arachnoid granulations into the blood or along cranial and spinal nerve roots into regional lymph nodes (Davson et al., 1987). Meningeal macrophages and the glia limitans, composed of astrocytic foot processes and a parenchymal basement membrane, impede the spread of pathogens from the CSF space into the brain tissue.

Bacteria can cross the blood–brain and blood–CSF barrier by (a) destruction of the endothelial cell layers (e.g., by pneumococcal pneumolysin), (b) traversal in between the cells of the barriers by disruption of the tight junctions, and (c) transcytosis, an intracellular transport route designed to transport molecules and vesicles through cells from the apical to basolateral side (Tenenbaum et al., 2009; Iovino et al., 2013). The adhesion of *Escherichia coli*, which reaches the brain by transcytosis, to membrane receptors of the cerebrovascular endothelium triggers a cascade of host cell signal transduction pathways resulting in host cell actin cytoskeleton rearrangements. This process involves host actin binding proteins and signaling molecules (e.g., Rac-1) and microbial determinants (e.g., IbeA and OmpA; Maruvada and Kim, 2012). *Streptococcus pneumoniae* preferentially adheres to the subarachnoid vessels, and only at the later stages of infection interacts with the endothelium of the choroid plexus (Iovino et al., 2013).

We hypothesize that the entry of a pathogen into the CNS probably is not uncommon. Yet, in an immunocompetent host the vast majority of pathogens, which eventually reach the brain tissue, are either eliminated or controlled in a latent form by the immune cells of the brain parenchyma, in particular, the microglial cells.

The immune defense of the CNS has been compared with a medieval castle. The blood–brain and blood–CSF barriers serve as the outer walls of the castle. The castle moat is represented by the CSF space. The second wall is represented by the glia limitans and resident macrophages. Inside the castle, i.e., the CNS parenchyma, “the royal family of sensitive neurons resides” protected by (micro)glial cells (Engelhardt and Coisne, 2011). Evidence for protective and reparative functions of microglial cells in the CNS has been found in diverse neurologic diseases, particularly in Alzheimer’s disease, stroke and excitotoxic brain injury (Anrather et al., 2011; Naert and Rivest, 2011; Woo et al., 2012). The beneficial aspects of the immune response in the nervous system are beginning to be appreciated and their potential as pharmacologic targets in neurologic disease is being explored (Graber and Dhib-Jalbut, 2009). In a mouse model of Alzheimer’s disease, repeated systemic injections of monophosphoryl lipid A, a LPS-derived Toll-like receptor 4 (TLR4) agonist that exhibits immunomodulatory properties at non-pyrogenic doses induced a potent phagocytic response by microglia, reduced the amyloidβ load in the brain and enhanced cognitive function (Michaud et al., 2013).

The density of microglial cells in brain tissue depends on the brain region. The healthy mouse brain contains an average of approximately 7000 cells/μl, i.e., the same order of magnitude as the density of leukocytes in the blood (calculated from Lawson et al., 1990 assuming a volume of the adult mouse brain of 0.5 ml). Microglia are the most abundant immune cells of the CNS. In their “resting” state, they continuously survey their environment with highly mobile processes (Nimmerjahn et al., 2005; Raivich, 2005). “Microglia are not notorious miscreants lurking in the CNS to harm neurons on any occasion. They are not placed there simply as a risk factor” (Hanisch, 2013). We are convinced that they are the key players to eliminate or at least control the replication of pathogens, which have entered the brain and spinal cord despite the fortifications surrounding the nervous tissue.

Statement: in healthy individuals, the protection of the CNS against infections relies both on the integrity of the blood–CSF and blood–brain barrier and on resident phagocytes, in particular microglial cells and perivascular and meningeal macrophages.

## PATHOPHYSIOLOGICAL ASPECTS OF ACUTE OR CHRONIC CNS INFECTIONS

Bacterial meningitis, meningoencephalitis, and brain abscess are life-threatening diseases with a high incidence in neonates, infants and in the immunocompromised and elderly. Besides classical pathogens (*S. pneumoniae, Neisseria meningitides, Haemophilus influenzae*) the causative agents of CNS infections in persons with an impaired immune system are other bacteria including Gram-negative aerobic rods, group B streptococci, *Nocardia* spp. and *Listeria monocytogenes* (Bouadma et al., 2006; Cabellos et al., 2008, 2009; Gaschignard et al., 2011; Lubart et al., 2011). Immunocompromised patients also are susceptible to meningitis and encephalitis caused by a variety of fungi, most frequently *Cryptococcus neoformans* in AIDS patients and *Aspergillus* spp. in patients on glucocorticoids and/or immunosuppressants. In bacterial and fungal meningitis, pathogens probably frequently cross the choroid plexus (i.e., the blood–CSF barrier; Tenenbaum et al., 2009), whereas in encephalitis and brain abscess, they often cross the cerebrovascular endothelium (i.e., the blood–brain barrier). In meningitis, encephalitis, and brain abscess, the causative pathogens also can enter the brain through a skull defect or along (thrombosed) vessels crossing the skull.

Many viruses have a propensity to cause latent infections and persist in cells of the central or peripheral nervous system. The majority of these viruses belong to the family of *Herpesviridae* (Traylen et al., 2011): herpes simplex virus (HSV)-1, HSV-2, varicella zoster virus (VZV), Epstein–Barr virus (EBV), cytomegalovirus (CMV), human herpesvirus (HHV)-6 and HHV-7. VZV, the most frequent viral cause of a reactivated infection of the nervous system (Herpes zoster, encephalitis, meningitis, myelitis, vasculitis), principally persists in neurons, and only occasionally in other cells of the CNS (Cohen, 2007). Whereas the incidence of HSV-1 encephalitis apparently is not increased in the immunocompromised host, the strongest risk factor for the development of VZV reactivation is age because of the age-related natural decline in cellular immunity to VZV. Furthermore, VZV, CMV, HSV-2, and HHV-6 and -7 typically cause reactivated infections in immunosuppressed individuals.

Despite antiretroviral therapy (ART), HIV infection is responsible for cognitive dysfunction and neurodegeneration through persistent viral replication in the CNS, inflammation and release of neurotoxic compounds from infected and/or activated macrophages/microglia (Cross et al., 2011). Because of the poor penetration of many antiretroviral drugs across the blood–brain and blood–CSF barrier, the CNS frequently is a site of continued HIV replication even when the viral load in blood is below the detection limit (Letendre et al., 2008; Nau et al., 2010; van Lelyveld et al., 2010).

Of the family of *Polyomaviridae*, JC virus causes progressive multifocal leukencephalitis (PML). The incidence of PML is <0.3 per 100,000 persons/year in the general population. In persons with an immune defect, either by an underlying disease or by the administration of monoclonal antibodies such as natalizumab, rituximab, efalizumab, and infliximab, or other immunosuppressants in the treatment of autoimmune or malignant disease the incidence is increased (Bellizzi et al., 2013) to 2.4 cases per 1000 persons/year in HIV-infected individuals without combination antiretroviral therapy (cART) and to 2.1 cases per 1000 patients/year in multiple sclerosis (MS) patients treated with natalizumab (Hirsch et al., 2013). Because natalizumab blocks α4-integrin-dependent lymphocyte entry into the brain, not the overall cellular immunodeficiency but the failure of the brain’s immune surveillance is considered responsible for the development of PML (Hirsch et al., 2013). JCV DNA was detected in oligodendrocytes, astrocytes and cerebellar granular cell neurons of the brains of humans without PML. The most common site for viral latency was cortical oligodendrocytes (65% of the samples analyzed). Immunocompromised patients more frequently harbored JCV DNA in cerebellar granular cell neurons than immunocompetent patients (Bayliss et al., 2012). This indicates that JCV DNA is present in cells of the human brain without clinical symptoms of PML and supports the hypothesis that reactivation of latent brain JCV may be central to disease pathogenesis (Bayliss et al., 2011, 2012).

The obligate intracellular parasite *Toxoplasma gondii* can infect and replicate within mammalian or avian cells including those residing in the brain. Circulating white blood cells, particularly dendritic cells and macrophages, are intracellularly infected and allow the parasite to spread hematogeneously to the brain and muscle (Kamerkar and Davis, 2012). *In vitro*, approximately 30% of the microglial cells compared to 10% of neurons and astrocytes were intracellularly infected with *T. gondii* (Lüder et al., 1999). *In vitro*, microglial cells and astrocytes were able to inhibit parasite replication upon activation (Chao et al., 1993). Traditionally, latent infections in humans were assumed to be largely asymptomatic, but recently behavioral abnormalities including schizophrenia have been linked with latent *T. gondii* infections (Webster, 2007). In HIV-infected individuals receiving no effective antiretroviral therapy, cerebral toxoplasmosis became a major complication and an AIDS-defining disease. When the host’s cerebral immune response weakens, parasite tissue cysts rupture and release bradyzoites which convert to rapidly dividing tachyzoites and cause *T. encephalitis* (Sullivan et al., 2009).

Statement: few typical pathogens only are able to overcome the immune defense of the healthy brain (e.g., *S. pneumoniae*, *N. meningitidis*, and *H. influenzae* causing acute meningitis and HSV-1 causing acute encephalitis). Conversely, in the immunocompromised host a wide spectrum of pathogens can cause acute, subacute or chronic CNS infections. The example of *T. gondii* illustrates that latent, principally well-controlled CNS infections even in immunocompetent hosts may lead to behavioral abnormalities.

## RESISTANCE OF THE HEALTHY BRAIN AGAINST INFECTIONS

The resistance of the brain to infections can be studied by direct injection of rapidly multiplying pathogens in a small volume into the brain parenchyma. With this experimental approach, the immune response in the first hours determines whether the host organism survives or eventually succumbs to an infection. Since circulating leukocytes generally need several hours to migrate into the central nervous compartments (Ernst et al., 1983), the ability of the host to eliminate invaded pathogens in the first hours depends on the local immune defense, i.e., the activity of microglial cells.

**FIGURE 1:**
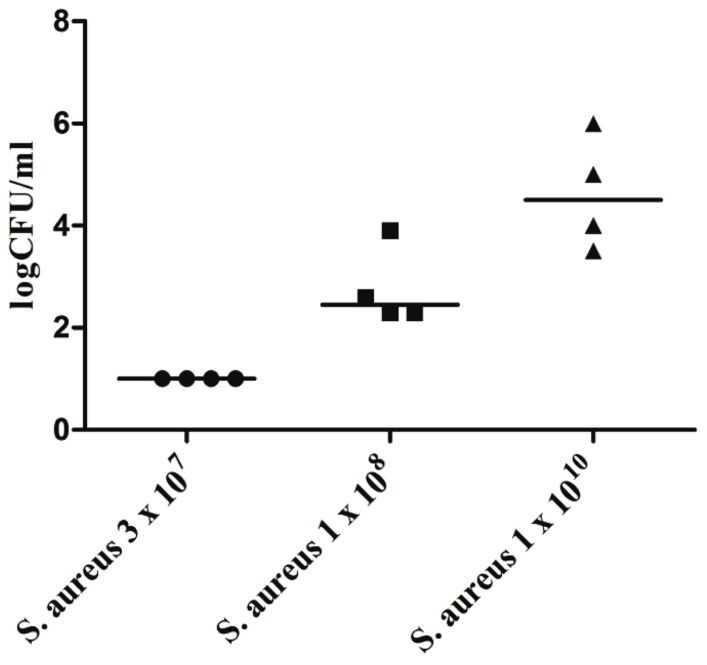
**Bacterial concentrations of *Staphylococcus aureus* American Type Culture Collection (ATCC) 29213 in cerebellar homogenates (*n* = 4 animals per inoculum) 168 h after intracerebral infection with different inocula of *S. aureus* under anesthesia.** Horizontal bars indicate median values, the detection limit was 10 colony-forming units (CFU)/ml (Marija Djukic, unpublished data). Please note that all mice were able to clear an inoculum of 3 × 10^7^ bacteria within 1 week.

For a variety of highly virulent pathogens, the healthy brain possesses a remarkable resistance to infection, e.g., the brain of an immunocompetent young mouse can clear up to 3 × 10^7^ colony-forming units (CFU) of *Staphylococcus aureus* injected into the forebrain without developing brain abscess or meningitis (**Figure 1**). To overcome the local defense of the mouse against this pathogen and reliably produce brain abscesses in experimental conditions, *S. aureus* had to be embedded into agarose beads (Kielian and Hickey, 2000).

**FIGURE 2:**
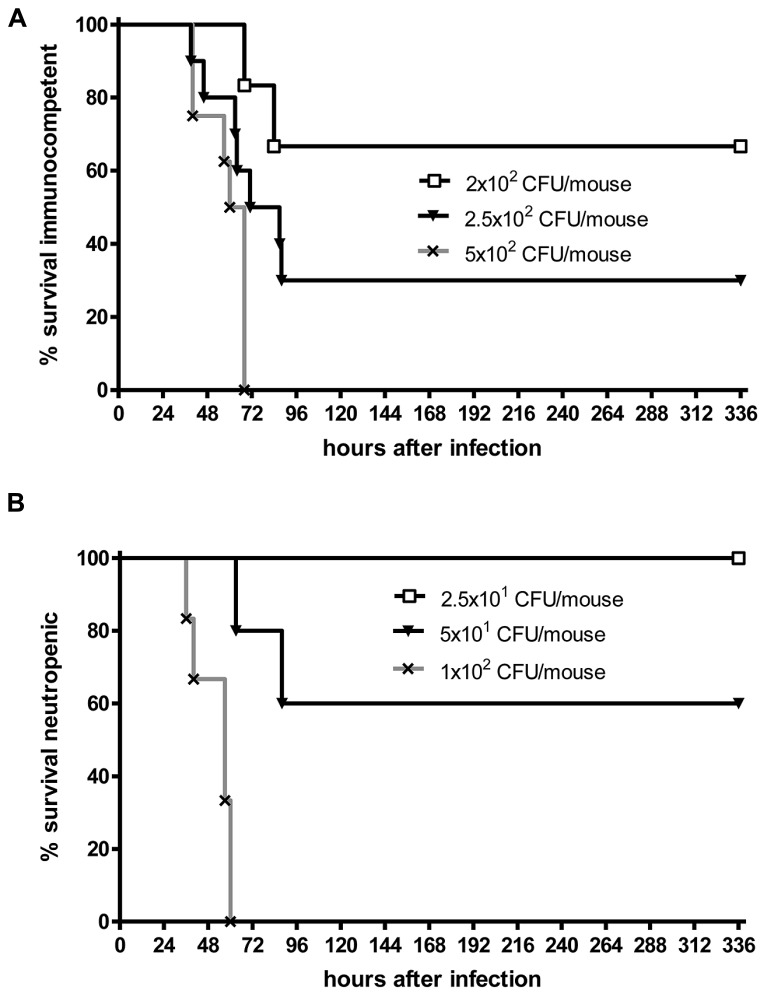
**Kaplan–Meier survival curves after intracerebral injection of different numbers of *Streptococcus pneumoniae* D39 (kindly provided by Prof. Dr. Sven Hammerschmidt, University of Greifswald, Germany). (A)** C57Bl/6 immunocompetent mice, **(B)** C57Bl/6 mice rendered neutropenic with 50 μg of an anti-Ly-6G monoclonal antibody (1A8 clone). Meningitis was induced by bacterial inoculation into the right frontal neocortex under anesthesia. Please note that 70% of immunocompetent mice survived an intracerebral injection of 200 colony-forming units (CFU). Conversely, all neutropenic mice were killed by an intracerebral inoculum of 100 CFU in less than 3 days suggesting that microglia need the cross-talk with granulocytes across the blood–brain barrier to function properly (Sandra Ribes, unpublished data).

**FIGURE 3:**
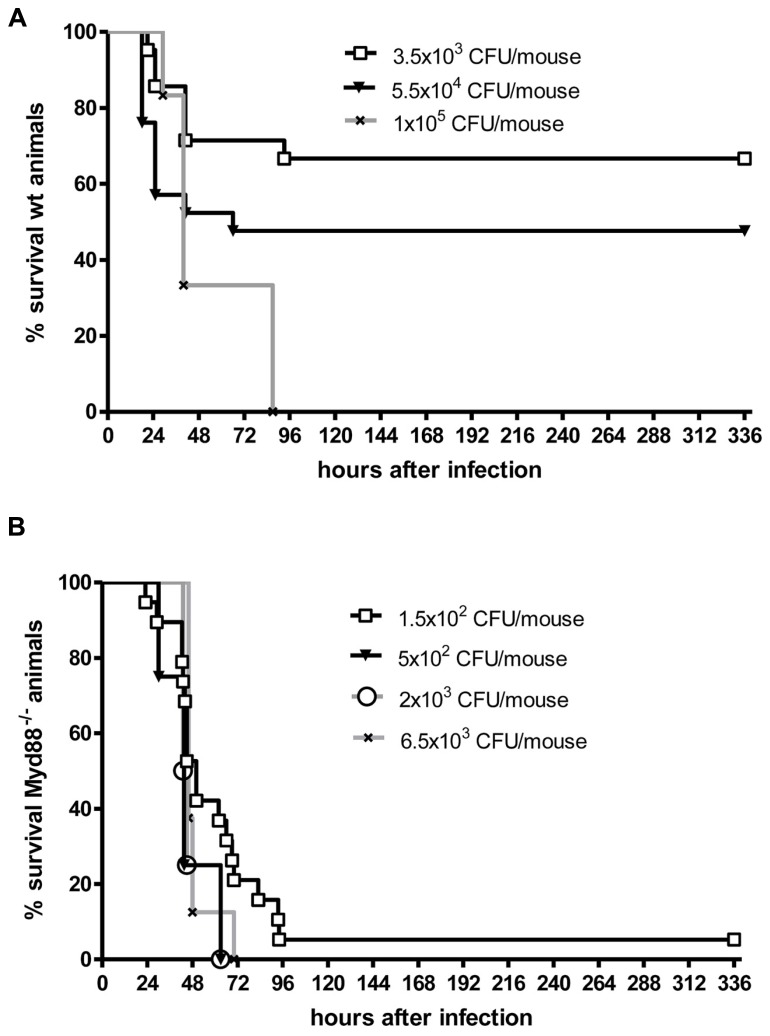
**Kaplan–Meier survival curves from (A) C57Bl/6J wild-type (wt) mice and (B) C57Bl/6J MyD88^-/-^ mice following intracerebral injection of different numbers of *Escherichia coli* K1 (kindly provided by Dr. Gregor Zysk, Düsseldorf).** Meningitis was induced by bacterial inoculation into the right frontal neocortex under anesthesia. Please note that 70% of immunocompetent mice survived an intracerebral injection of 3500 colony-forming units (CFU). Conversely, over 90% of MyD88^-/-^ mice were killed by an intracerebral inoculum of 150 CFU in less than 4 days suggesting that MyD88 as a central signaling molecule of the innate immune system is essential for the inactivation of bacteria by parenchymal microglia and macrophages (Sandra Ribes, unpublished data).

Even when *S. pneumoniae*, the most frequent agent causing community-acquired bacterial meningitis, is injected into the murine neocortex in a low number, depending on the injected inoculum and the mouse strain used some animals are capable to overcome the infection without development of meningitis. The inoculum size necessary to induce lethal meningitis with *E. coli*, a pathogen predominantly causing meningitis in newborns, infants, old, or immunosuppressed people, lies between the inocula necessary to induce *S. pneumoniae* and *S. aureus* meningoencephalitis (**Figures 2** and **3**).

Although white blood cells are not at the site of intracerebral infection in the first hours, granulocytopenia leads to a strong increase of the susceptibility of mice to an intracerebral injection of *S. pneumoniae* or *E. coli* (**Figures 2** and **3**). This observation underlines the importance of the cross-talk between resident immune cells, the vascular endothelium and circulating leukocytes (Perry et al., 2003) for the proper functioning of microglial cells. In experimental septicaemia-induced *S. pneumoniae* meningitis, microglia appeared to sense bacterial adhesion to the endothelial cells and were activated immediately, before meningitis developed (Iovino et al., 2013).

Microglia as part of the mammalian innate immune system express germ line-encoded pattern recognition receptors (PRRs) that are crucial in the recognition of pathogens. Toll-like receptors (TLRs), RIG-like receptors (RLRs), and nucleotide-binding oligomerization domain-leucine rich repeat containing (NLR) proteins serve as PRRs that recognize different microbial structures, so-called pathogen-associated molecular patterns (PAMPs). TLR signaling and the resulting transcriptional activation of immune response genes requires the adaptor molecule myeloid differentiation factor 88 (MyD88), except for TLR3 signaling. Intact TLR → MyD88 signaling was essential for the survival in the acute phase of infection and for control of bacterial replication in both intracerebrally inoculated *E. coli* K1 (Ribes et al., 2013) and intracisternally injected *S. pneumoniae* (Koedel et al., 2004). In our experience, Myd88^-/-^ mice rapidly succumbed to *E. coli* meningoencephalitis even at a very low inoculum size (**Figure 3**). After oral infection with *T. gondii*, MyD88 was essential in establishing the protective host response in the CNS (Torres et al., 2013). *T. gondii*, an intracellular protozoan parasite, establishes a latent chronic infection primarily in the brain after replication of the parasite (tachyzoite form) in various organs during the acute stage of infection. While other organs (e.g., liver, lungs, heart, and small intestine) were not affected, in Myd88^-/-^ mice mortality was caused by severe toxoplasmic encephalitis which correlated with low numbers of CD8^+^ T cells and a significantly higher infiltration of the brain with CD11b^+^ and F4/80^+^ cells compared to Myd88^+^^/^^+^ control mice (Torres et al., 2013). F4/80^+^ cells were together with CD11c^-^ and CD11b^+^ cells responsible for parasite dissemination to the brain (Courret et al., 2006; Torres et al., 2013). Macrophage/microglial activation was observed all over different brain regions of *T. gondii*-infected Myd88^-/-^, and only deficient mice showed numerous clusters of ameoboid microglial cells (Torres et al., 2013).

TLR3 is a sensor for viral double-stranded RNA (dsRNA) which signals through the Toll–interleukin-1 (IL-1) receptor (TIR)-domain-containing adaptor inducing IFN-β (TRIF). A protective role of the TLR3-TRIF-mediated pathway was reported in response to Poliovirus (PV), the causative agent of poliomyelitis (Abe et al., 2012). TLR3 and TRIF signaling pathways inhibited PV replication in non-neural tissues thereby influencing the viral invasion of the CNS. TRIF-deficient mice (trif^lps2^) were not more susceptible to intracerebral *E. coli* K1 infection than wild-type mice of the same age and background (Ribes et al., 2013).

Statement: the resistance of the CNS of healthy hosts to infection as assessed by direct injection of pathogens into the brain tissue is higher than often perceived. The infection resistance relies on the ability of microglial cells and resident macrophages to phagocytose and kill pathogens, which is strengthened by the cross-talk with circulating immune cells.

## MICROGLIA CAN BE STIMULATED TO PHAGOCYTOSE AND KILL BACTERIA AND FUNGI

*In vitro*, unstimulated microglia phagocytosed *E. coli* and *S. pneumoniae* at a low rate. Phagocytosis was stimulated by TLR2, 3, 4, and 9 agonists and by the nucleotide-binding oligomerization domain-containing protein 2 (NOD2) agonist muramyl dipeptide (Ribes et al., 2009, 2010a,b, 2012). These agents also increased intracellular bacterial killing. The presence of a bacterial capsule as one important virulence factor decreased the rate of phagocytosis of *E. coli* and *S. pneumoniae* by one order of magnitude compared to unencapsulated strains (Ribes et al., 2009, 2010a,b, 2012). Agonists of the innate immune system also enhanced the phagocytosis of *Cryptococcus neoformans* (Redlich et al., 2013). The increase of phagocytosis by stimulation of microglial cells via agonists of receptors of the innate immune was accompanied by a release of NO and proinflammatory cytokines.

Phagocytosis of *Mycobacterium tuberculosis* by microglial cells can be inhibited by antibodies against CD14, which together with TLR4 forms the LPS receptor (Peterson et al., 1995). Microglial cells also can phagocytose the predominantly intracellular pathogen *L. monocytogenes*. After bacterial ingestion, microglia appeared to act as Trojan horses, transporting and releasing the phagocytosed bacteria inside the brain tissue after systemic infection (Serbina et al., 2008; Remuzgo-Martínez et al., 2013). To our knowledge, intracellular killing of mycobacteria and *L. monocytogenes* by microglial cells has not been studied. Among human monocytes, two subsets indistinguishable by the expression of cell surface markers involved in the phagocytosis of microbes were observed: approximately 75% of all monocytes were not very active in phagocytosing *L. monocytogenes*, but restricted intracellular growth. Approximately 25% could ingest a large number of bacteria and permitted intracellular growth of *L. monocytogenes* (Zerlauth et al., 1996). Monocytes which were able to kill *L. monocytogenes*, secreted more TNFα than those who allowed intracellular replication of bacteria (Zerlauth et al., 1996). *In vitro*, microglias were able to phagocytose and inhibit the growth of *T. gondii* in a NO-dependent manner, which was stimulated by IFNγ and LPS. In contrast to microglial cells, uptake of *T. gondii* into astrocytes was parasite-driven, and astrocytes were unable to inhibit multiplication of tachyzoites suggesting that astrocytes may provide a safe harbor for *T. gondii* (Peterson et al., 1993).

For the phagocytosis and intracellular killing of many pathogens, data on microglial cells are incomplete. The contribution of monocytes to the control of other pathogens able to cause CNS infections including fungi is compiled in a recent review (Serbina et al., 2008).

In *E. coli* experimental meningitis, pre-treatment with the TLR9 agonist cytosine-guanine oligodeoxynucleotide 1668 (CpG) strongly increased survival of neutropenic C57Bl6 mice. The protective effect was associated with long-term increased levels of IL-12/IL-23p40 in serum and spleen. CpG-treated neutropenic mice had reduced bacterial concentrations in brain and spleen 42 h after infection (Ribes et al., 2014). Although the administration of CpG caused sickness behavior and the release of proinflammatory cytokines, no long-term neurological abnormalities were noted in surviving CpG-treated mice. This work indicates that systemic immunostimulation with a TLR9 agonist can not only protect against systemic bacterial infections but also against an intracerebral bacterial challenge (Ribes et al., 2014). In microglial-neuronal co-cultures, stimulation of microglia by Pam_3_CSK_4_ (TLR2), LPS (TLR4), and CpG (TLR9) caused injury to the neurons starting at the axons (Iliev et al., 2004; Schütze et al., 2012). Therefore, the approach may entail the risk of inducing collateral damage to the nervous tissue.

Statement: microglia can be stimulated by a variety of compounds to phagocytose and kill pathogens *in vitro*. Preliminary data suggest that systemic administration of these compounds can also increase the resistance of the brain to infections. This approach, however, may entail the risk of inducing collateral damage to the nervous tissue

## MICROGLIAL CYTO- AND CHEMOKINE RELEASE DEPENDS ON THE PROINFLAMMATORY STIMULUS AND THE SUBSET OF MICROGLIAL CELLS ACTIVATED

The microglial response upon contact with products of infectious agents or synthetic analogs of them is not uniform: in primary microglial cultures from newborn mice, incubation of microglia with agonists of the TLR2 (0.1 μg/ml Pam_3_CSK_4_), TLR4 (0.01 μg/ml LPS), and TLR9 (1 μg/ml CpG) at the lowest concentrations inducing maximum NO release caused a comparable release of TNFα (Ribes et al., 2009). *In vitro* and *in vivo*, however, synthesis of TNFα is not a common feature of all microglia, but restricted to a specific subset. The proportion of TNFα-producing microglia increased from approx. 30% in neonatal to 75% in young adult microglial cells (Scheffel et al., 2012; Hanisch, 2013). The synthesis of CCL3 (macrophage inflammatory protein 1α, MIP-1α) was restricted to a rather small subpopulation, and most of the CCL3-synthesizing cells belonged to the (larger) population of TNFα producers (Scheffel et al., 2012; Hanisch, 2013).

Unlike TNFα, stimulation of microglia with the same TLR agonists at equal potencies resulted in a divergent release of CXCL1. Pam_3_CSK_4_ at 0.1 μg/ml was the stimulus that induced higher CXCL1 concentrations in the cell culture supernatants compared to 0.01 μg/ml LPS and 1 μg/ml CpG (Ribes et al., 2009). While stimulation with the TLR2 agonist Pam_3_CSK_4_ led to a predominant release of IL6, incubation with the TLR4 ligand LPS produced the highest levels of CCL5 (RANTES) (Ribes et al., 2010b). The subset distribution of the cells releasing these cyto- and chemokines remains to be determined. Our data, however, demonstrate that microglial cells do not respond uniformly to infectious stimuli, but are able to mount a nuanced reaction.

Human innate immune cells stimulated by TLR agonists selective for TLR7 or TLR8 also did not react in a uniform way: TLR7 agonists directly activated plasmacytoid dendritic cells and, to a lesser extent, monocytes. On the contrary, TLR8 agonists directly activated myeloid dendritic cells, monocytes, and monocyte-derived dendritic cells. TLR7-selective agonists were more potent than TLR8-selective agonists at inducing IFN-inducible protein and IFN-inducible T cell alpha chemoattractant from human mononuclear cells. Conversely, TLR8 ligands were more effective than TLR7 agonists at inducing TNFα, IL-12 and MIP-1α (Gorden et al., 2005). In microglia, the TLR4 co-receptor CD14 played an important role in controlling the profiles of cyto/chemokine production. Dependent on the TLR4 agonist (different classes of LPS, fibronectin) and the activation of co-receptors, distinct signaling routes are activated leading to a modulation of response profiles of key cytokines (Regen et al., 2011). Hence, for the immune response of microglial cells it also appears of importance which TLR and which co-receptor(s) are stimulated.

Statement: unlike previously thought, an accumulating body of evidence demonstrates the diversity of microglial cells. This opens avenue to selectively stimulate sub-populations responsible for the defence against pathogens without stimulating sub-populations which are responsible for collateral damage to the nervous tissue.

## THE EFFICIENCY OF MICROGLIA TO PREVENT CNS INFECTIONS DEPENDS ON CO-OPERATION WITH CIRCULATING IMMUNE CELLS

Microglia and CNS macrophages cross-talk with circulating blood cells by several mechanisms (Perry et al., 2003): (a) the bi-directional passage of circulating cytokines or pro-inflammatory pathogen compounds through leaks of the blood–brain barrier (physiologically in the circumventricular organs, under pathological conditions at lesion sites with an impaired blood–brain barrier), (b) the activation of endothelial cells and perivascular macrophages either by circulating or brain-derived compounds, (c) via the vagus nerve. CD11b^+^Ly-6G^+^Ly-6C^int^ granulocytes and CD11b^+^Ly-6G^-^Ly-6C^high^ monocytes in the bloodstream were required for the control of intracerebrally injected *E. coli* K1 (Ribes et al., 2013). Animals that received intraperitoneally an anti-Gr-1 antibody (clone RB6-8C5) showed an earlier and higher mortality than anti-IgG_2b_-treated mice (92.3 vs. 11.1%, *P* = 0.0002). Depletion of CD11b^+^Ly-6G^+^Ly-6C^int^ granulocytes from the systemic circulation by intraperitoneal injection of the anti-Ly-6G antibody (clone 1A8) also had an influence on the survival of *E. coli* K1 infected mice (mortality 59.2% compared to 34.6% in anti-IgG_2a_-treated animals, *P* = 0.049). We hypothesize that after intracerebral bacterial infection the primary immune response determines whether the infected organism will survive without antibiotic treatment or will eventually succumb to the infection. This is supported by the course of the clinical score in mice. All animals which develop serious symptoms of infection eventually die, unless they are treated with antibiotics. Animals which eventually survive either show no signs of infection or are mildly lethargic in the first hours after infection and then recover. Immediately after inoculation of bacteria, before bacteria start to multiply, resident phagocytes as the primary line of defence have the opportunity to phagocytose and kill the inoculum. Data from experimental *S. pneumoniae* meningitis models suggest that after bacterial inoculation granulocytes need approx. 12 h (e.g., Ernst et al., 1983) to migrate into the CSF, i.e., in the first hours after infection the resident phagocytes are on their own. When the inoculum is high enough to overcome the primary immune defence of resident phagocytes, invading granulocytes are not able to control multiplication of bacteria in the intracranial compartments. Granulocyte invasion therefore represents a futile secondary immune response in this condition. In the CSF of rabbits rendered leukopenic by nitrogen mustard, the growth rate of *S. pneumoniae* was slightly increased (mean generation time 60 vs. 67 min), and ultimate bacterial density in the CSF was slightly higher than in immunocompetent rabbits, i.e., leukocytes did not effectively slow or limit the growth of pneumococci in the CSF *in vivo* (Ernst et al., 1983). For these reasons, the strong detrimental effect of neutropenia on the resistance of the brain to intracerebral *E. coli* infection was unexpected and can be only explained by the cross-talk of resident phagocytes and circulating leukocytes across the blood–brain and blood–CSF barrier (Ribes et al., 2013).

Statement: the protective action of microglia against infections critically depends on the cross-talk with circulating granulocytes and monocytes in the first hours, before circulating leukocytes enter the brain and CSF. For this reason, immunosuppressive measures affecting all or specific subsets of circulating leukocytes probably bear the risk of also affecting microglial function by impairing this cross-talk.

## PHAGOCYTOSIS AND KILLING OF PATHOGENS BY MICROGLIA DOES NOT NECESSARILY REQUIRE NITRIC OXIDE OR CYTOKINE RELEASE

**FIGURE 4:**
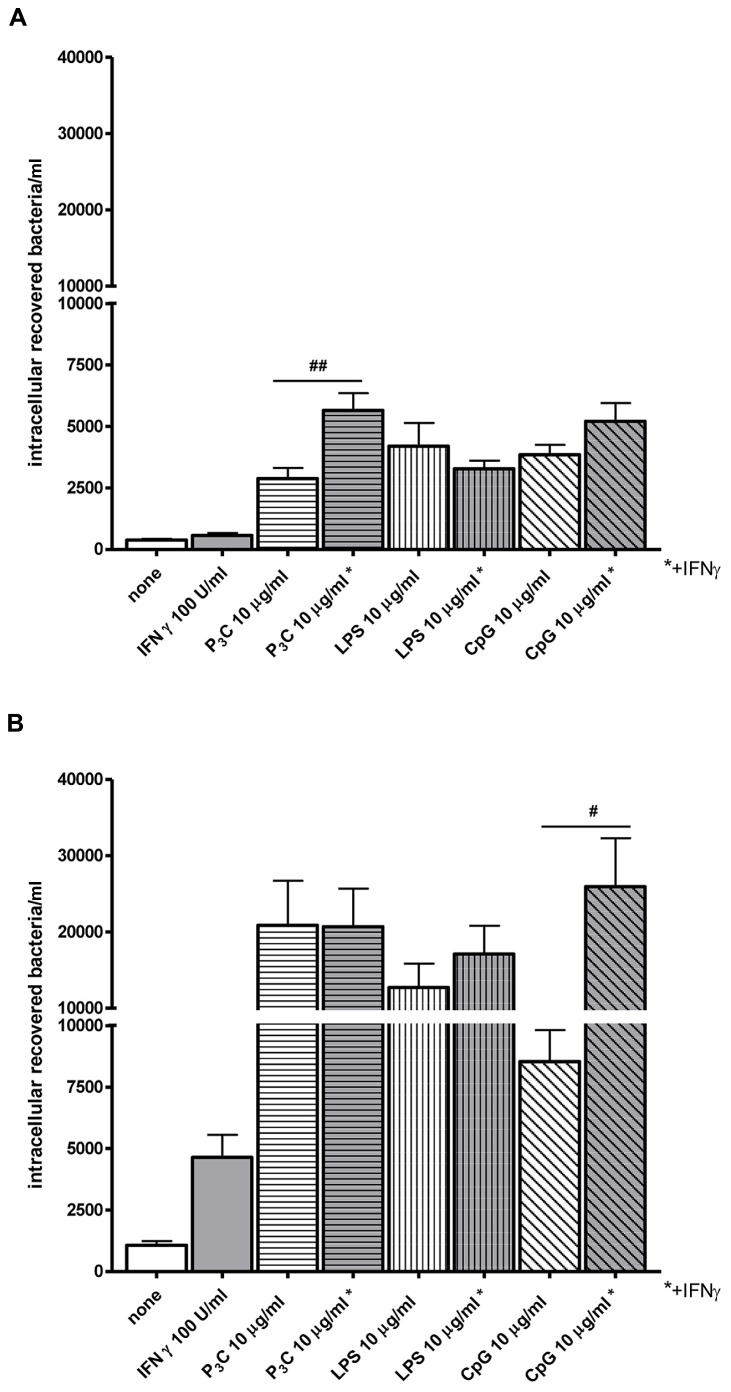
**Effect of interferonγ (IFNγ) on bacterial phagocytosis by Toll-like receptor-stimulated primary microglia. (A)** 30 min and **(B)** 90 min of phagocytosis of *E. coli* DH5α by microglial cells after 24 h of stimulation with the TLR agonists: 0.1 μg/ml Pam_3_CSK_4_ (P3C), 0.01 μg/ml LPS and 1 μg/ml CpG alone or in combination with 100 U/ml IFNγ. After stimulation, cells were washed and bacteria were added for different time periods (30 and 90 min). Then, gentamicin (200 μg/ml) was added for 1 h to kill extracellular bacteria. Thereafter, bacteria were lysed in 0.1 ml of distilled water. The number of ingested bacteria was determined by quantitative plating of the cell lysates after the different incubation intervals. Data are shown as recovered bacteria (CFU) per well [mean ± SEM (error bars)]. Data were analyzed using one-way ANOVA followed by Bonferroni’s multiple comparison test (^#^*P* < 0.05; ^##^*P* < 0.01; Sandra Ribes, unpublished data).

**FIGURE 5:**
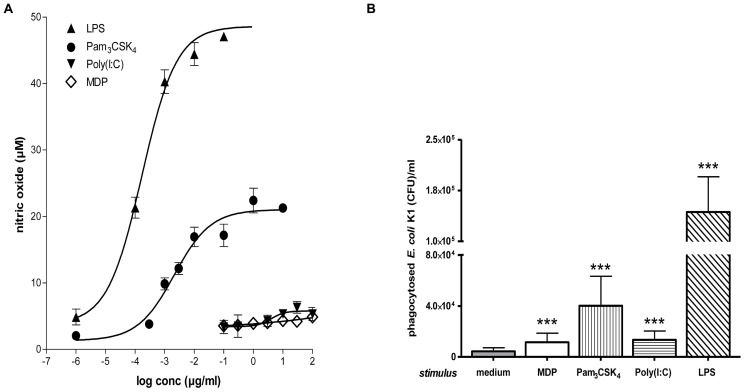
**Microglial NO release and phagocytosis of bacteria. (A)** Stimulation with TLR2 or 4 agonists induced stronger microglial nitric oxide (NO) release than stimulation with a TLR3 agonist or muramyl dipeptide (MDP). Dose–response relations after 24 h treatment with various concentrations of agonists of TLR1/2 (Pam_3_CSK_4_), TLR3 [poly(I:C)], TLR4 (LPS), and NOD2 (MDP; Ebert et al., 2005; Ribes et al., 2009; Ribes et al., 2012). **(B)** Phagocytosis of *E. coli* K1 strain by microglial cells after 24 h of stimulation with the agonists at the lowest concentration inducing maximum NO release: 0.1 μg/ml Pam_3_CSK_4_, 10 μg/ml poly(I:C), 0.01 μg/ml LPS, and 10 μg/ml MDP (means ± SD, ^***^p < 0.001 vs. medium-treated cells); Student’s *t* test and correction for repeated testing by the Bonferroni–Holm method. Please note that although stimulation with poly(I:C) and MDP released low amounts of NO, both compounds led to a substantial increase of bacterial phagocytosis (Figure reproduced from Ribes et al., 2012, with permission of the publisher).

Bacterial clearance by phagocytes of the central nervous compartments apparently does not always require the presence of interferonγ (IFNγ) and nitric oxide (NO): in experimental *S. pneumoniae* meningitis, IFNγ^-/-^ mice showed reduced gene expression of NO synthase, but bacterial clearance was enhanced in IFNγ^-/-^ compared to wild-type infected animals (Mitchell et al., 2012). *In vitro*, the addition of 100 U/ml IFNγ did not increase the uptake of an unencapsulated strain of *E. coli* by TLR4-stimulated microglia while it moderately increased the bacterial ingestion by TLR2- and TLR9-stimulated cells (**Figure 4**). In microglial cultures pre-stimulated with different agonists of the innate immune system, NO release and phagocytosis of bacteria do not strongly correlate (Ribes et al., 2012; **Figure 5**).

At present, it is unclear whether all microglial cells or only a fraction of them is involved in the phagocytosis and inactivation of pathogens. Myelin uptake under pathophysiological conditions apparently is executed by a fraction of microglia (Scheffel et al., 2012; Hanisch, 2013). For this reason, it is probable that a subgroup of microglial cells bears phagocytic activity against pathogens (Hanisch, 2013). Selective stimulation of the activity of the subgroup(s) of microglial cells, which phagocytose pathogens, appears a feasible goal of future research.

Statement: since NO release probably is a main cause of collateral damage to nervous tissue after microglial activation, the finding that elimination of pathogens does not necessarily depend on NO release encourages the search for stimulants which increase pathogen clearance without inducing damage to the nervous tissue.

## ANTIBIOTIC TREATMENT: REDUCING THE RELEASE OF PROINFLAMMATORY COMPOUNDS OF PATHOGENS IN ORDER TO PREVENT EXCESSIVE MICROGLIAL ACTIVATION

Injury and mortality of bacterial meningitis is caused by the joint action of multiplying bacteria and released bacterial products, the local immune response of the brain and by granulocytes and monocytes invading the subarachnoid space and nervous tissue from the blood (Nau and Brück, 2002; Mook-Kanamori et al., 2011; Nau et al., 2013).The contribution of these individual mechanisms depends on the pathogen and the host’s immune response. Microglial cells stimulated by bacterial products can kill neurons *in vitro* (Iliev et al., 2004). Reduction of the amount of proinflammatory/toxic pathogen-derived products avoids overstimulation of resident and migrating immune cells including microglia and thereby protects the nervous tissue. For this reason, it appears desirable to keep the concentrations of proinflammatory products of pathogens in the central nervous tissue low during the whole course of an infection including its treatment. This aspect of microglial stimulation has been most extensively studied in bacterial meningitis, but probably is of importance also for other CNS infections, particularly for those with a high pathogen load.

In clinical practice, a favorable outcome depends on rapid antibiotic treatment after hospital admission (Auburtin et al., 2006). Although many antibiotics commonly used in the management of bacterial meningitis cause bacterial lysis and subsequent release of proinflammatory or directly cytotoxic compounds, rapid antibiotic treatment stops bacterial replication and production of bacterial products and therefore on the long run reduces the concentrations of proinflammatory/toxic bacterial products in the CNS (Stuertz et al., 1998; Spreer et al., 2003). *In vitro*, temporarily the pneumolysin release was higher in ceftriaxone-treated *S. pneumoniae* compared to untreated *S. pneumoniae* cultures. After 3 h, however, the pneumolysin release during spontaneous growth exceeded the release after initiation of ceftriaxone treatment. In particular, a strong increase of extracellular pneumolysin was observed when untreated pneumococci reached the end of the logarithmic growth phase (Spreer et al., 2003).

In contrast to the classical view considering bactericidal and bacteriolytic synonyms, the cidal and the lytic effects of an antibiotic do not necessarily coincide. With cell-wall active antibacterials, in particular beta-lactam antibiotics, lytic and cidal action are tightly linked. Bactericidal antibiotics acting by the inhibition of RNA or protein synthesis or DNA replication (rifamycins, macrolides, clindamycin, ketolides, and with some limitations also quinolones) circumvent or at least delay bacterial lysis (for review, see Nau and Eiffert, 2002, Nau et al., 2013). In animal models of bacterial meningitis, rifampicin, clindamycin and daptomycin reduced inflammation, mortality, neuronal injury or/and neurological long-term sequelae compared to the standard therapy with beta-lactam antibiotics (Nau et al., 1999; Gerber et al., 2003; Böttcher et al., 2004; Grandgirard et al., 2007, 2012; Spreer et al., 2009; Barichello et al., 2013; Nau et al., 2013).

Statement: the reduction of potentially deleterious pathogen-derived compounds by rapid initiation of an effective antibiotic therapy or by choosing compounds which do not release large amounts of pathogen products is a promising strategy to avoid overstimulation of microglial cells and decrease neuronal injury.

## PLEIOTROPIC COMPOUNDS INHIBITING MICROGLIAL FUNCTION

The role of corticosteroids in infections of the CNS has been debated for decades.

*In vitro*, dexamethasone inhibited the release of TNFα and IL-1β by microglia after exposure to LPS (Forshammar et al., 2013). After dexamethasone exposure, pronounced transcriptional effects were observed in microglia, where 257 genes were differentially expressed. The majority of these genes were related to the immune function (Jenkins et al., 2013). Conversely, corticosteroids can delay the production of myelin. In high-purity cell cultures, however, oligodendrocyte lineage cells were not influenced by exposure to dexamethasone. Untreated microglial cell cultures showed a branching ramified morphology indicative of the “resting” state. Dexamethasone exposure dramatically reduced cell densities, and thereafter many cells showed a rounded appearance. Corticosteroid-treated microglia may demonstrate an impaired ability to migrate toward and phagocytose cells/synapses and myelin debris and probably thereby indirectly affects myelination (Jenkins et al., 2013). Whether corticosteroids also inhibit phagocytosis and intracellular inactivation of pathogens by microglia, remains to be studied.

Dexamethasone as an adjunct to antibiotic treatment in the adult rabbit model of *S. pneumoniae* and in the infant rat models of *Streptococcus* group B and *S. pneumoniae* meningitis aggravated apoptotic neuronal injury in the hippocampal dentate gyrus and impaired long-term learning capacity of surviving rats in the Morris water maze compared to rats treated with an antibiotic only (Leib et al., 1996, 2003; Zysk et al., 1996; Spreer et al., 2006; Nau et al., 2013). There is, however, no evidence of an aggravation of human hippocampal injury in bacterial meningitis by adjunctive dexamethasone (Brouwer et al., 2013; Nau et al., 2013). Data from clinical trials at present point to a beneficial effect of corticosteroids as an adjunct to antibiotic therapy in community-acquired acute bacterial and Tuberculous meningitis (Thwaites et al., 2009; Brouwer et al., 2013). In other CNS infections (e.g., cerebral toxoplasmosis, brain abscess, HSV encephalitis), glucocorticoids should be used only in the presence of a life-threatening brain edema.

Cytostatics such as mitoxantrone and cyclophosphamide and the antimetabolites methotrexate and azathioprine act on a variety of proliferating cells and as immunosuppressants probably also inhibit proliferation and function of microglial cells (e.g., Vairano et al., 2004; Li et al., 2012). New agents used for the treatment of MS also have pleiotropic effects including a direct influence on microglial activity. Fingolimod inhibits autoreactive lymphocytes from infiltrating the CNS, but also downregulates the production of the pro-inflammatory cytokines TNFα, IL-1β, and IL-6 by activated microglia (Noda et al., 2013).

Statement: the long-term use of pleiotropic compounds affecting microglial function increases the risk of CNS infections. In community-acquired bacterial meningitis, dexamethasone as an adjunct to antibiotic treatment at present represents the standard therapy to inhibit the systemic inflammatory and the local microglial response of the host. Corticosteroids or other pleiotropic immunosuppressive compounds probably are not the ideal agents for this purpose, because they affect the ability of microglia to clear pathogens. This is of particular importance, when antiinfectives are not able to kill all causative pathogens.

## COMPOUNDS STIMULATING PHAGOCYTOSIS BY MICROGLIAL CELLS WITHOUT INDUCING AN INFLAMMATORY REACTION

Palmitoylethanolamide (PEA) is a small endogenous lipid (molecular mass 299.5 g/mol) which is widely present in cells including microglia (Muccioli and Stella, 2008), tissues and body fluids. It has analgesic, anticonvulsant, neuroprotective, antipyretic and anti-inflammatory properties. Its actions depend mainly on the peroxisome proliferator-activated receptor (PPAR)α, but it also is a ligand of the transient receptor potential vanilloid-1 (TRPV1) and the orphan G-protein coupled receptor GPR55 (De Petrocellis et al., 2001; LoVerme et al., 2005, 2006; Ryberg et al., 2007; Esposito and Cuzzocrea, 2013). In a murine Theiler’s virus model of chronic MS, treatment with PEA (5 mg/kg) between days 60 and 70 post-infection resulted in a strong improvement of motor deficits caused by a reduction of microglial activation observed in untreated mice (Loría et al., 2008).

**FIGURE 6:**
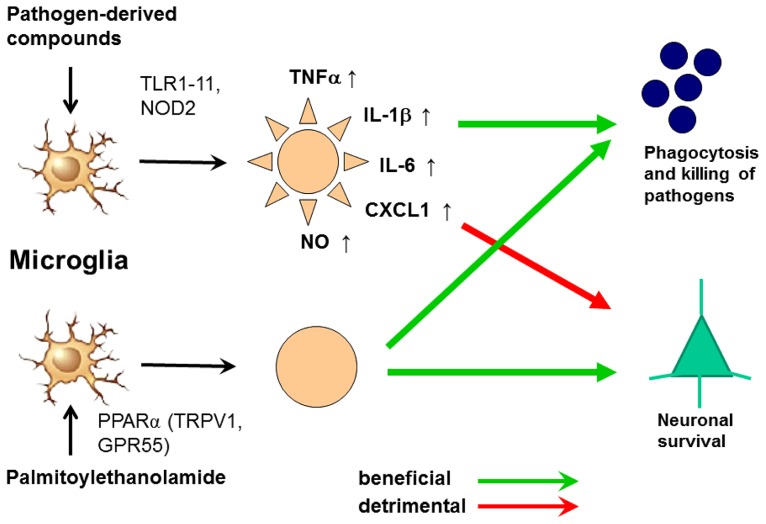
**Activation of microglia by Toll-like receptor (TLR) agonists and by palmitoylethanolamide (PEA).** The activation by both ways leads to an increase of phagocytosis and intracellular killing of pathogens. Stimulation of one or several TLR or nucleotide-binding oligomerization domain-containing protein 2 (NOD2) receptors causes the release of proinflammatory products from microglial cells causing neuronal injury in microglial-neuronal co-cultures and probably also in vivo. PEA also increases phagocytosis and intracellular killing of pathogens. To our knowledge, it does not release proinflammatory mediators. For this reason we hypothesize that it will not cause collateral neuronal injury. PEA probably acts via the peroxisome proliferator-activated receptor (PPAR)α, but also is a ligand of the transient receptor potential vanilloid-1 (TRPV1) and the orphan G-protein coupled receptor GPR55 (De Petrocellis et al., 2001; LoVerme et al., 2005, 2006; Ryberg et al., 2007; Esposito and Cuzzocrea, 2013).

In spite of its anti-inflammatory properties, 30 min pre-treatment with PEA stimulated phagocytosis of *S. pneumoniae* (EC_50_ 5.9 nM) and *E. coli* (EC_50_ 23 nM) by microglial cells *in vitro*. It was not toxic to microglial cells up to a concentration of 1000 nM. Unlike pre-stimulation with TLR and NOD2 agonists, the PEA-mediated increase of microglial bacterial uptake was not accompanied by a release of pro-inflammatory cyto-/chemokines [TNFα, IL-6, and CXCL1 (KC)], avoiding the risk of concomitant neuronal injury (Redlich et al., 2012). Preliminary data suggest that PEA also decreases the susceptibility of the brain to intracerebral injection of bacteria (Sandra Redlich, unpublished data). From 1969 to 1979, PEA was tested under the brand name Impulsin^R^ (SPOFA United Pharmaceutical Works, Prague, Czechoslovakia) in prophylactic and therapeutic clinical trials (five in adults, one in children): it reduced the incidence and severity of acute respiratory infections and influenza (Kahlich et al., 1979; Keppel Hesselink et al., 2013). More than 3600 patients received PEA at daily doses from 600 to 1800 mg, and no severe adverse effects were reported (Kahlich et al., 1979; Keppel Hesselink et al., 2013).These properties illustrate that PEA is a true immunomodulator and not an immunosuppressant and make PEA a promising agent to enhance the resistance of the brain against infection without carrying the risk of inducing neuronal injury (**Figure 6**). This effect may be of clinical value both in preventing bacterial CNS infections in high-risk groups and in reducing the invasion of pathogens into brain tissue in manifest meningeal infections.

Glatiramer acetate also increased phagocytosis *in vitro* (Pul et al., 2011, 2012): the ingestion of fluorescent beads was greater in monocytes from MS patients treated with glatiramer acetate than in those from healthy controls or non-treated MS patients. Only monocytes co-expressing CD16 were observed to phagocytose, and addition of IL-10 did not decrease phagocytosis (Pul et al., 2012). Moreover, in primary rat microglia glatiramer acetate promoted the phagocytosis of fluorescent latex beads and increased IL-10 secretion, whereas it decreased the release of TNFα and did not affect NO release (Pul et al., 2011). Phagocytosis of bacteria, however, was not studied. Glatiramer acetate, a random polymer of four amino acids found in myelin basic protein (glutamic acid, lysine, alanine, tyrosine) as a relatively large hydrophilic compound (molecular mass approx. 600 g/mol) in contrast to PEA probably crosses the blood–brain and blood–CSF barrier to a small extent only. It remains to be studied, whether the glatiramer acetate CNS concentrations are high enough to influence the phagocytic activity of microglia.

Dimethyl fumarate is a small lipophilic compound (molecular mass 144.1 g/mol). Treatment of HIV-infected human monocyte-derived macrophages with dimethyl fumarate attenuated HIV replication in a dose-dependent manner, as determined by reverse transcriptase concentrations in the culture supernatants. Dimethyl fumarate also inhibited NF-κB translocation and the release of TNFα from phytohemagglutinin-activated macrophages and reduced HIV-induced TNFα release from macrophages (Cross et al., 2011; Gill and Kolson, 2013). Dimethyl fumarate together with other esters of fumaric acid has been marketed for several years as an antipsoriasis drug. Lymphocytopenia and eosinophilia are frequent side effects, and the compound appears to increase the risk of PML, Kaposi sarcoma and nocardiosis (manufacturer’s information, http://www.akdae.de/Arzneimittelsicherheit/RHB/20130625.pdf).

Statement: the data obtained with PEA and, with limitations, glatiramer acetate and dimethyl fumarate underline that the search for modulators instead of inhibitors of microglial activity appears promising.

## CONCLUSION

Phagocytosis of pathogens by microglial cells can be stimulated by agonists of receptors of the innate immune system. The use of this signaling pathway to increase the resistance of the brain against infections entails the risk of inducing collateral damage to the nervous tissue. As a consequence of microglial diversity, it appears possible to identify compounds that increase pathogen uptake and elimination and thereby may contribute to the protection of the brain without a concomitant measurable proinflammatory effect. In this context, PEA, an endogenous lipid which had been tested clinically in the 1970s and apparently decreased the incidence of respiratory infections without severe side effects, appears to be most promising. Based on *in-vitro* data, glatiramer acetate and dimethyl fumarate both used for the treatment of MS appear as other potential candidates.

## Conflict of Interest Statement

The authors declare that the research was conducted in the absence of any commercial or financial relationships that could be construed as a potential conflict of interest.
